# Drug-induced peripheral nerve palsy: a real-world study based on FAERS data from 2004 to the third quarter of 2024

**DOI:** 10.3389/fphar.2025.1691263

**Published:** 2026-01-08

**Authors:** Yingjie Li, Xueliang Yi, Nengwei Yu

**Affiliations:** 1 Department of Neurology, Sichuan Provincial People’s Hospital, University of Electronic Science and Technology of China, Chengdu, China; 2 Department of Emergency, Clinical Medical College & Affiliated Hospital of Chengdu University, Chengdu University, Chengdu, China

**Keywords:** adverse events, disproportionality analysis, FAERS, peripheral nerve palsy, pharmacovigilance

## Abstract

**Background:**

Peripheral nerve palsy is a prevalent clinical condition that significantly impairs patients’ quality of life. To advance clinical practice and mitigate the risk of drug-induced peripheral nerve palsy, this study aimed to identify adverse drug reaction signals related to peripheral nerve palsy through data mining of the FDA Adverse Event Reporting System (FAERS). Timely detection of high-risk medications provides a crucial basis for enhancing the safety of clinical drug use.

**Methods:**

Adverse events (AEs) related to peripheral nerve palsy between 2004 and the third quarter of 2024 were extracted from the FAERS database. To identify potential drug safety signals associated with peripheral nerve palsy, four established pharmacovigilance statistical methods were employed: the Reporting Odds Ratio (ROR), Proportional Reporting Ratio (PRR), Multi-Item Gamma Poisson Shrinker (MGPS), and Bayesian Confidence Propagation Neural Network (BCPNN).

**Results:**

A total of 5,787 reports of drug-associated peripheral nerve palsy adverse events involving 1,141 drugs were identified in this analysis. Among these, natalizumab had the highest number of reported cases, followed by interferon beta-1a and dalfampridine. The most commonly implicated drug classes were antineoplastic drugs and immunosuppressants. A total of 30 drugs exhibited positive risk signals, of which 19 met the criteria for a positive signal across all four analytical algorithms. Notably, both the ROR and BCPNN methods indicated that bupivacaine, dalfampridine, natalizumab, minocycline, and ocrelizumab are among the high-risk drugs associated with peripheral nerve palsy.

**Conclusion:**

This study identified that several medications, including bupivacaine, dalfampridine, natalizumab, minocycline, and ocrelizumab, are significantly associated with an increased risk of peripheral nerve palsy. These findings contribute to a deeper understanding of drug safety profiles, support the promotion of rational medication use, and provide valuable insights to inform clinical decision-making.

## 1 Introduction

Peripheral nerve palsy is a frequently observed manifestation of peripheral neuropathy. It occurs when peripheral nerves sustain damage to a significant degree, impairing their ability to conduct nerve impulses effectively. As a result, muscles fail to receive proper neural signals, leading to symptoms such as muscle weakness and palsy. Peripheral neuropathy itself refers to structural or functional abnormalities in the peripheral nerves—including sensory, motor, and autonomic nerves—arising from a variety of causes. These causes may include metabolic disorders, exposure to toxins, ischemia (reduced blood flow), inflammation, physical trauma or mechanical compression, presence of tumors, and genetic factors (McKinley and Perkins, 2019). Clinically, peripheral neuropathy presents with symptoms such as limb numbness, pain, reduced sensation, and diminished muscle strength. There are both overlaps and distinctions between nerve palsy and neuropathy. Neuropathy is a more general term that encompasses a wide range of pathological conditions affecting the peripheral nerves. These can involve damage to the nerve fibers themselves, demyelination, and axonal degeneration. In contrast, nerve palsy specifically describes the clinical manifestation where there is a loss or reduction in muscle movement function due to impaired nerve function. While neuropathy and nerve palsy often occur together, not all cases of neuropathy progress to nerve palsy. Typically, nerve palsy represents a more severe stage in the progression of peripheral neuropathy or results from specific, often more localized, causative factors.

The clinical manifestations of peripheral nerve palsy include: ① impaired muscle strength due to disrupted innervation; ② reduced or absent muscle tone, resulting in muscle flaccidity and lack of resistance to external tension; ③ diminished or absent tendon reflexes; ④ noticeable muscle atrophy; ⑤ possible presence of muscle fasciculations or muscle fiber tremors. The diagnosis of nerve palsy is established based on clinical signs, physical examination findings, and neurophysiological assessments (McKinley and Perkins, 2019; Mbuya, 2006; Castelli et al., 2020).

In recent years, with the increasing prevalence of medication use and the continuous introduction of new pharmaceutical agents, drug-induced adverse reactions have garnered growing attention. Among these, drug-induced peripheral neuropathy has emerged as a relatively common and often debilitating condition (Vilholm et al., 2014; Jones et al., 2020; Peltier and Russell, 2002). The most severe manifestations of this condition include nerve palsy, which frequently develop either during the course of treatment or shortly after its completion. If not promptly recognized and managed, nerve palsy can result in significant functional impairments. Early detection and appropriate therapeutic intervention are crucial for minimizing the risk of long-term disability and preventing irreversible damage to nerve function. Consequently, identifying the specific medications associated with the development of nerve palsy has become a pivotal aspect of clinical diagnosis and treatment strategies.

It has been demonstrated that certain medications may elevate the risk of neuropathy (Bełtowski et al., 2009; Zhang, 2021; Kyncl et al., 2023; Li et al., 2020), such as isoniazid (Sankar et al., 2024), ethambutol (Sudhakar et al., 2025), nelarabine (Shakeel et al., 2025), and vincristine (Mauermann and Staff, 2025), among others. However, there remains a notable absence of large-scale, systematic studies specifically aimed at evaluating the association between various medications and the occurrence of nerve palsy. This gap in knowledge not only complicates clinicians’ ability to make well-informed decisions regarding drug prescriptions but also restricts comprehensive assessments of drug safety. The FDA Adverse Event Reporting System (FAERS) represents the largest publicly accessible database for the spontaneous reporting of adverse drug events. It aggregates data contributed by healthcare professionals, consumers, and pharmaceutical manufacturers (Zhou et al., 2023; Yin et al., 2022), and plays a crucial role in disseminating information about the potential risks associated with medications. By analyzing data from the FAERS database, researchers can identify potential correlations between specific drugs and adverse events, thereby offering valuable insights into clinical drug safety. Previous studies have utilized this database to investigate drug-induced conditions such as macular degeneration (Chen et al., 2025), hypoglycemia (Li et al., 2024), allergic reactions (Yu et al., 2021), and Parkinson’s-like events (Wang et al., 2025), thereby providing important references to guide clinical decision-making.

The objective of this study was to evaluate the risk of drug-induced peripheral nerve palsy through a disproportionality analysis utilizing data from the FAERS spanning from 2004 to the third quarter of 2024. By identifying medications that are most strongly associated with an increased risk of nerve palsy, the study aims to furnish clinicians with enhanced safety information to guide drug administration. This approach is intended to support the rational use of medications and, ultimately, to mitigate associated risks, thereby offering valuable insights for improving clinical practice in the future.

## 2 Methods

### 2.1 Data sources

**FIGURE 1:**
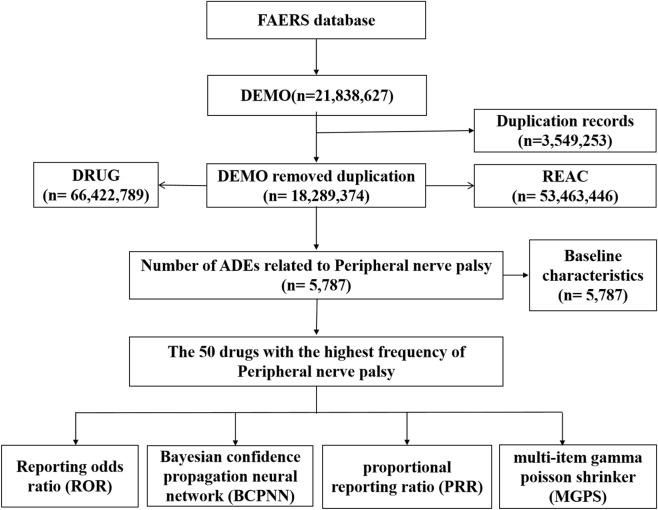
Flowchart of data acquisition.

This retrospective pharmacovigilance study was based on the FAERS database (https://fis.fda.gov/extensions/FPD-QDE-FAERS/FPD-QDE-FAERS.html). FAERS is a database designed to support post-marketing surveillance programmes for drugs and therapeutic biologics, including all AE information and medication error information collected by the FDA. All data in the database is voluntarily submitted by healthcare professionals and consumers (Yu et al., 2021), and we can find a lot of information from it, such as demographic characteristics, drug information, clinical outcome information, and more. We conducted this retrospective pharmacovigilance analysis using the FAERS database. FAERS is a compendium of adverse drug event (ADE) reports that allows researchers to detect signals and quantify associations between drugs and ADEs (Zhou et al., 2023; Yin et al., 2022). FAERS database is updated quarterly (Cui et al., 2023). Due to the nature of data updates, duplicate reporting is inevitable in FAERS. Therefore, we set the search timeframe from 1 January 2004 to 30 September 2024 and removed duplicates to improve the reliability of the results based on the following criteria recommended by the FDA: if CASEIDs were the same, the most recent FDA_DT was selected; if CASEIDs and FDA_DTs were the same, the higher PRIMARYID was selected (Cui et al., 2023). After data preprocessing, we obtained 18,289,374 DEMO reports, 66,422,789 DRUG cases, and 53,463,446 REAC records, and 5,787 reports of drug-related peripheral nerve palsy adverse events (Figure 1). A total of 1,141 drugs have been associated with adverse reactions to peripheral nerve palsy, and our study analyses reported the top 50 drugs in terms of the number of adverse events that occurred. Because FAERS is a publicly accessible, completely anonymised dataset that does not include personally identifiable information, the study adheres to ethical guidelines and does not require Institutional Review Board approval, in line with the FDA’s policy of ensuring data privacy and confidentiality.

### 2.2 Study design (identification of target adverse event reports)

In the FAERS database, the Medical Dictionary for Regulatory Activities (MedDRA) is used to code the various ADR messages into standardised medical terms called Preferred Terminology (PT). Standardised MedDRA queries (SMQs) are built-in tools in MedDRA, consisting of a series of PTs indicating similar medical conditions, designed to help retrieve cases of interest from MedDRA coding databases and optimise Adverse Drug Reactions (ADR) signal detection and assessment. SMQs provides two types of searches to identify target cases, wide range search and narrow range search. A wide range search contains all PT that may represent the condition or area of interest, while a narrow range search contains only PT that is closely related to the condition or area of interest. In this study, we looked up peripheral nerve palsy according to the preferred terminology of the International Medical Dictionary of Regulatory Activities (MedDRA version 26.1), and we listed the common peripheral nerve palsies, see Supplementary Table S1.

### 2.3 Data mining algorithms and data analysis

Disproportionality analysis is a data mining algorithm used to quantify ADR signals in large pharmacovigilance databases. We used four established signal detection algorithms: reported ratio of ratios (ROR), proportional reporting ratio (PRR), Bayesian confidence propagation neural network (BCPNN), and multi-item gamma Poisson shrinker (MGPS) (Chen L. et al., 2024; Li et al., 2025). Based on the classic 2-on-2 contingency Table (Table 1), disproportionality analysis can be used to analyze the difference between the occurrence frequency and background frequency of target drugs and target AEs, thereby establishing a statistical association between drugs and AEs. In this study, a combination of frequency method and Bayes method was used to mine ADR signals, and the lower limit of 95% CI > 1, PRR ≥ 2, χ^2^ ≥ 4, IC025 > 0, EBGM > 1, EBGM05 > 2 were met, one of which was defined as an ADR signal. Table 1 is a quadruple table of disproportionality analyses. Table 2 shows the calculation formula and Criteria, and the larger the ROR and BCPNN, the stronger the signal, indicating the greater the correlation between the target drug and ADE. In this study, R version 4.4.2 was used for data acquisition, processing, and analysis.

**TABLE 1:** Two-by-two contingency table for disproportionality analyses.

Item	Target AEs	Other AEs	Total
Drugs	a	b	a+b
Other drugs	c	d	c+d
Total	a+c	b+d	a+b+c+d

Abbreviation: AEs, adverse events; a, number of reports containing both the target drug and target adverse drug reaction; b, number of reports containing other adverse drug reaction of the target drug; c, number of reports containing the target adverse drug reaction of other drugs; d, number of reports containing other drugs and other adverse drug reactions.

**TABLE 2:** Four major algorithms used for signal detection.

Algorithms	Equation	Criteria
ROR	ROR = ad/b/c	Lower limit of 95% CI > 1, N ≥ 3
	95% CI = e^ln(ROR)±1.96(1/a+1/b+1/c+1/d)^0.5^	
PRR	PRR = a(c+d)/c/(a+b)	PRR ≥ 2, χ^2^ ≥ 4, N ≥ 3
	χ^2^ = [(ad-bc)^2](a+b+c+d)/[(a+b)(c+d)(a+c)(b+d)]	
BCPNN	IC = log_2_a(a+b+c+d)(a+c)(a+b)	IC025 > 0
	95% CI = E(IC) ± 2V(IC)^0.5	
MGPS	EBGM = a(a+b+c+d)/(a+c)/(a+b)	EBGM05 > 2
	95%CI = e^ln(EBGM)±1.96(1/a+1/b+1/c+1/d)^0.5^	

Abbreviation: a, number of reports containing both the target drug and target adverse drug reaction; b, number of reports containing other adverse drug reaction of the target drug; c, number of reports containing the target adverse drug reaction of other drugs; d, number of reports containing other drugs and other adverse drug reactions. 95% CI, 95% confidence interval; N, the number of reports; χ^2^, chi-squared; IC, information component; IC025, the lower limit of 95% CI of the IC; E(IC), expectation(s) for the IC; V(IC), the variance of IC; EBGM, empirical Bayesian geometric mean; EBGM05, the lower limit of 95% CI of EBGM.

## 3 Result

### 3.1 Demographic information pertaining to AE reports

**FIGURE 2:**
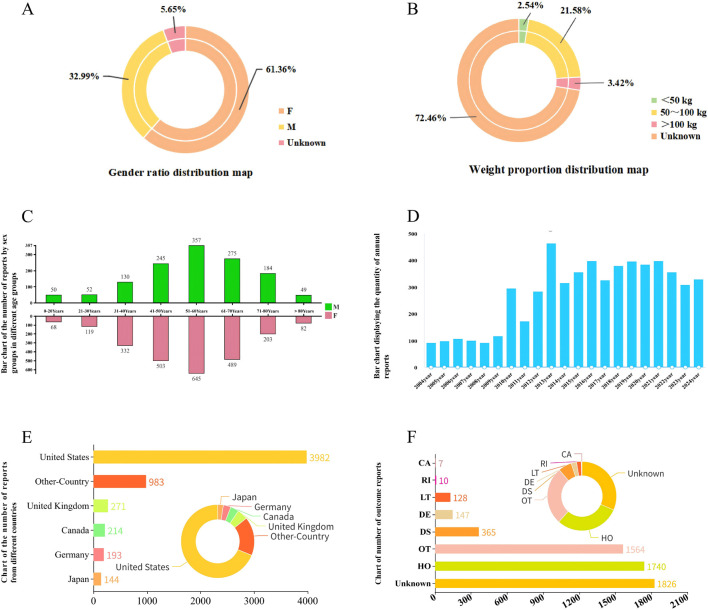
**(A)** Gender ratio distribution map. **(B)** Weight proportion distribution map. **(C)** Bar chart of the number of reports by sex groups in different age groups. **(D)** Bar chart displaying the quantity of annual reports. **(E)** Chart of the number of reports from different countries. **(F)** Chart of number of outcome reports.

From January 2004 to September 2024, we conducted a comprehensive analysis of a total of 21,838,627 adverse event reports extracted from the FAERS database. Among these reports, 5,787 were specifically associated with peripheral nerve palsy. At baseline, the patient population exhibited a notable gender disparity, with female patients accounting for 61.36% of the reports, significantly outnumbering male patients, who made up 32.99% (Figure 2A). This gender imbalance was consistently observed across all analyzed age groups (Figure 2C). Based on the demographic data available in the FAERS database, the most frequently reported age group among individuals experiencing peripheral nerve palsy was between 51 and 60 years old (Figure 2C). Additionally, the majority of AEs were reported within the weight range of 50–100 kg (Figure 2B). Over the study period, the annual number of reported peripheral nerve palsy cases demonstrated a steady upward trend from 2004 to 2013. Following the peak incidence during that timeframe, the number of reported cases plateaued and remained relatively stable in subsequent years (Figure 2D). Geographically, the distribution of reported cases showed that the United States accounted for the largest proportion, contributing 68.81% of all reports. This was followed by the United Kingdom (4.68%), Canada (3.70%), Germany (3.34%), and Japan (2.49%) (Figure 2E). In terms of the occupational distribution of reporters, consumers represented the largest group, comprising 3,097 (53.52%), followed by health practitioners with 2,462 (42.54%). In terms of adverse event outcomes, most adverse events were Hospitalisation-Initial or Prolonged (1,740, 30.07%) and other serious conditions (1,564, 27.03%) (Figure 2F; Table 3).

**TABLE 3:** Demographic information reported by AE in FAERS.

Characteristics	Reports, n	Reports, %
Gender		
F	3,551	61.36%
M	1,909	32.99%
Unknown	327	5.65%
Weight		
<50 kg	147	2.54%
50∼100 kg	1,249	21.58%
>100 kg	198	3.42%
Unknown	4,193	72.46%
Age		
0–20 year	118	2.04%
21–30 year	171	2.95%
31–40 year	462	7.98%
41–50 year	748	12.93%
51–60 year	1,002	17.31%
61–70 year	764	13.20%
71–80 year	387	6.69%
>80 year	131	2.26%
Unknown	2,004	34.63%
Reported person		
CN	3,097	53.52%
Health professionals	2,462	42.54%
Unknown	228	3.94%
Reported countries (top five)		
United States	3,982	68.81%
United Kingdom	271	4.68%
Canada	214	3.70%
Germany	193	3.34%
Japan	144	2.49%
Other-Country	983	16.99%
Outcome		
CA	7	0.12%
DE	147	2.54%
DS	365	6.31%
HO	1,740	30.07%
LT	128	2.21%
OT	1,564	27.03%
RI	10	0.17%
Unknown	1,826	31.55%

CN, consumers; DE, death; LT, life-threatening; DS, disability; CA, congenital anomaly; RI, required intervention to prevent permanent impairment; HO, hospitalization-initial or prolonged; OT, other serious conditions; NR, not reported.

### 3.2 Drug screening and disproportionality analysis

**FIGURE 3:**
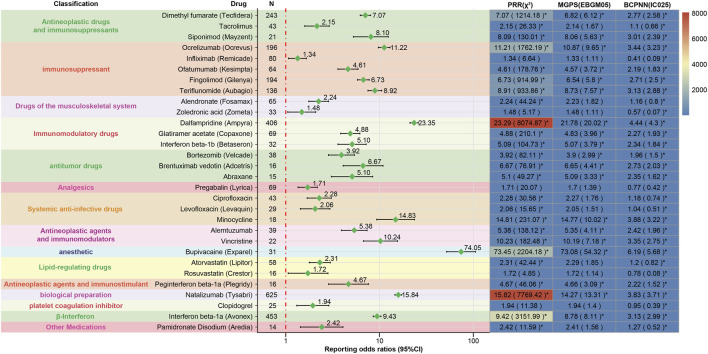
A heatmap classifies positive signal drugs by mechanism of action, with darker colors showing higher signal values and greater risk of drug-related peripheral nerve palsy. PRR, proportional reported ratio; MGPS, multi-item gamma poisson shrinker; BCPNN, bayesian confidence propagation neural network.

Our study examined the 50 drugs that had the highest number of adverse events reported. These 50 drugs were categorized based on their mechanisms of action as follows: Antineoplastic drugs and immunosuppressants (9; 18.00%), Immunosuppressant (8; 18.00%), Drugs of the musculoskeletal system (4; 8.00%), Immunomodulatory drugs (3; 6.00%), antitumor drugs (3; 6.00%), Analgesics (3; 6.00%), Systemic anti-infective drugs (3; 6.00%), Antineoplastic agents and immunomodulators (2; 4.00%), anesthetic (2; 4.00%), Drugs of the cardiovascular system (2; 4.00%), Lipid-regulating drugs (2; 4.00%), Antidepressant drug (1; 2.00%), Antineoplastic agents and immunostimulant (1; 2.00%), Antipsychotic drug (1; 2.00%), biological preparation (1; 2.00%), Medications for Diabetes (1; 2.00%), platelet coagulation inhibitor (1; 2.00%), β-Interferon (1; 2.00%), Other Medications (2; 4.00%) (Table 4). Disproportionate analyses conducted using four algorithms identified 30 medications associated with positive risk signatures for peripheral nerve palsy. Among these, the five drugs with the highest number of reports were natalizumab (n = 625), interferon beta-1a (n = 453) and dalfampridine (n = 406), dimethyl fumarate (n = 243), and ocrelizumab (n = 196). Among the 30 drugs analyzed, 19 exhibited a positive signal across all four algorithms. Based on the ROR of each individual drug as an indicator of risk signal strength, the top five drugs are as follows: bupivacaine [ROR (95% CI):74.05 (51.95–105.54)], dalfampridine [ROR (95% CI):23.35 (21.11–25.83)], natalizumab [ROR (95% CI):15.84 (14.58–17.21)], Minocycline [ROR (95% CI):14.83 (9.33–23.56)], Ocrelizumab [ROR (95% CI):11.22 (9.73–12.94)]. This observation was subsequently visualized and is presented in Figure 3.

**TABLE 4:** Statistical values and distribution of drug-induced peripheral nerve palsy.

Drug	Number	Classification	ROR (95% Cl)	PRR (*X* ^2^)	MGPS (EBGM05)	BCPNN (IC025)
Dimethyl fumarate (Tecfidera)	243	Antineoplastic drugs and immunosuppressants	7.07 (6.22–8.04)^a^	7.07 (1214.18)^a^	6.82 (6.12)^a^	2.77 (2.58)^a^
Methotrexate	47	Antineoplastic drugs and immunosuppressants	0.83 (0.62–1.11)	0.83 (1.64)	0.83 (0.65)	−0.27 (−0.69)
Lenalidomide (Revlimid)	46	Antineoplastic drugs and immunosuppressants	0.6 (0.45–0.8)	0.6 (12.41)	0.6 (0.47)	−0.74 (−1.16)
Tacrolimus	43	Antineoplastic drugs and immunosuppressants	2.15 (1.59–2.91)^a^	2.15 (26.33)^a^	2.14 (1.67)	1.1 (0.66)^a^
Rituximab	35	Antineoplastic drugs and immunosuppressants	0.74 (0.53–1.03)	0.74 (3.26)	0.74 (0.56)	−0.44 (−0.92)
Bevacizumab (Avastin)	27	Antineoplastic drugs and immunosuppressants	1.26 (0.86–1.83)	1.26 (1.41)	1.26 (0.91)	0.33 (−0.22)
Siponimod (Mayzent)	21	Antineoplastic drugs and immunosuppressants	8.1 (5.27–12.43)^a^	8.09 (130.01)^a^	8.06 (5.63)^a^	3.01 (2.39)^a^
Ibrutinib (Imbruvica)	18	Antineoplastic drugs and immunosuppressants	0.79 (0.5–1.25)	0.79 (1)	0.79 (0.54)	−0.34 (−1)
Upadacitinib (Rinvoq)	16	Antineoplastic drugs and immunosuppressants	1.08 (0.66–1.76)	1.08 (0.09)	1.08 (0.72)	0.11 (−0.59)
Ocrelizumab (Ocrevus)	196	Immunosuppressant	11.22 (9.73–12.94)^a^	11.21 (1762.19)^a^	10.87 (9.65)^a^	3.44 (3.23)^a^
Infliximab (Remicade)	80	Immunosuppressant	1.34 (1.07–1.66)^a^	1.34 (6.64)	1.33 (1.11)	0.41 (0.09)^a^
Ofatumumab (Kesimpta)	64	Immunosuppressant	4.61 (3.6–5.9)^a^	4.61 (178.76)^a^	4.57 (3.72)^a^	2.19 (1.83)^a^
Secukinumab (Cosentyx)	13	Immunosuppressant	0.29 (0.17–0.51)	0.29 (22.06)	0.3 (0.19)	−1.76 (−2.53)
Fingolimod (Gilenya)	194	Immunosuppressant	6.73 (5.83–7.77)^a^	6.73 (914.99)^a^	6.54 (5.8)^a^	2.71 (2.5)^a^
Adalimumab (Humira)	164	Immunosuppressant	0.81 (0.69–0.94)	0.81 (7.53)	0.81 (0.71)	−0.3 (−0.53)
Teriflunomide (Aubagio)	136	Immunosuppressant	8.92 (7.53–10.58)^a^	8.91 (933.86)^a^	8.73 (7.57)^a^	3.13 (2.88)^a^
Etanercept (Enbrel)	77	Immunosuppressant	0.52 (0.42–0.65)	0.52 (33.46)	0.53 (0.44)	−0.92 (−1.25)
Alendronate (Fosamax)	65	Drugs of the musculoskeletal system	2.24 (1.76–2.86)^a^	2.24 (44.24)^a^	2.23 (1.82)	1.16 (0.8)^a^
Zoledronic acid (Zometa)	33	Drugs of the musculoskeletal system	1.48 (1.05–2.09)^a^	1.48 (5.17)	1.48 (1.11)	0.57 (0.07)^a^
Denosumab (Prolia)	20	Drugs of the musculoskeletal system	0.57 (0.37–0.89)	0.57 (6.38)	0.57 (0.4)	−0.8 (−1.43)
Teriparatide (Forteo)	16	Drugs of the musculoskeletal system	0.45 (0.28–0.74)	0.45 (10.66)	0.45 (0.3)	−1.14 (−1.84)
Dalfampridine (Ampyra)	406	Immunomodulatory drugs	23.35 (21.11–25.83)^a^	23.29 (8074.87)^a^	21.78 (20.02)^a^	4.44 (4.3)^a^
Glatiramer acetate (Copaxone)	69	Immunomodulatory drugs	4.88 (3.85–6.18)^a^	4.88 (210.1)^a^	4.83 (3.96)^a^	2.27 (1.93)^a^
Interferon beta-1b (Betaseron)	32	Immunomodulatory drugs	5.1 (3.6–7.21)^a^	5.09 (104.73)^a^	5.07 (3.79)^a^	2.34 (1.84)^a^
Bortezomib (Velcade)	38	Antitumor drugs	3.92 (2.85–5.39)^a^	3.92 (82.11)^a^	3.9 (2.99)^a^	1.96 (1.5)^a^
Brentuximab vedotin (Adcetris)	16	Antitumor drugs	6.67 (4.09–10.9)^a^	6.67 (76.91)^a^	6.65 (4.41)^a^	2.73 (2.03)^a^
Abraxane	15	Antitumor drugs	5.1 (3.07–8.46)^a^	5.1 (49.27)^a^	5.09 (3.33)^a^	2.35 (1.62)^a^
Pregabalin (Lyrica)	69	Analgesics	1.71 (1.35–2.17)^a^	1.71 (20.07)	1.7 (1.39)	0.77 (0.42)^a^
Rofecoxib (Vioxx)	32	Analgesics	1.26 (0.89–1.78)	1.26 (1.68)	1.26 (0.94)	0.33 (−0.17)
Oxycodone (Oxycontin)	18	Analgesics	0.4 (0.25–0.63)	0.4 (16.6)	0.4 (0.27)	−1.33 (−2)
Ciprofloxacin	43	Systemic anti-infective drugs	2.28 (1.69–3.07)^a^	2.28 (30.56)^a^	2.27 (1.76)	1.18 (0.74)^a^
Levofloxacin (Levaquin)	29	Systemic anti-infective drugs	2.06 (1.43–2.96)^a^	2.06 (15.65)^a^	2.05 (1.51)	1.04 (0.51)^a^
Minocycline	18	Systemic anti-infective drugs	14.83 (9.33–23.56)^a^	14.81 (231.07)^a^	14.77 (10.02)^a^	3.88 (3.22)^a^
Alemtuzumab	39	Antineoplastic agents and immunomodulators	5.38 (3.93–7.37)^a^	5.38 (138.12)^a^	5.35 (4.11)^a^	2.42 (1.96)^a^
Vincristine	22	Antineoplastic agents and immunomodulators	10.24 (6.73–15.56)^a^	10.23 (182.48)^a^	10.19 (7.18)^a^	3.35 (2.75)^a^
Bupivacaine (Exparel)	31	Anesthetic	74.05 (51.95–105.54)^a^	73.45 (2204.18)^a^	73.08 (54.32)^a^	6.19 (5.68)^a^
Sodium oxybate (Xyrem)	18	Anesthetic	0.81 (0.51–1.29)	0.81 (0.77)	0.81 (0.55)	−0.3 (−0.96)
Amlodipine	26	Drugs of the cardiovascular system	1.44 (0.98–2.12)	1.44 (3.55)	1.44 (1.05)	0.53 (−0.03)
Sacubitril Valsartan sodium (Entresto)	22	Drugs of the cardiovascular system	0.61 (0.4–0.92)	0.61 (5.63)	0.61 (0.43)	−0.72 (−1.32)
Atorvastatin (Lipitor)	58	Lipid-regulating drugs	2.31 (1.78–2.99)^a^	2.31 (42.44)^a^	2.29 (1.85)	1.2 (0.82)^a^
Rosuvastatin (Crestor)	16	Lipid-regulating drugs	1.72 (1.06–2.82)^a^	1.72 (4.85)	1.72 (1.14)	0.78 (0.08)^a^
Sertraline	19	Antidepressant drug	1.24 (0.79–1.95)	1.24 (0.9)	1.24 (0.85)	0.31 (−0.33)
Peginterferon beta-1a (Plegridy)	16	Antineoplastic agents and immunostimulant	4.67 (2.86–7.64)^a^	4.67 (46.06)^a^	4.66 (3.09)^a^	2.22 (1.52)^a^
Quetiapine (Seroquel)	29	Antipsychotic drug	1.03 (0.72–1.48)	1.03 (0.03)	1.03 (0.76)	0.04 (−0.49)
Natalizumab (Tysabri)	625	Biological preparation	15.84 (14.58–17.21)^a^	15.82 (7769.42)^a^	14.27 (13.31)^a^	3.83 (3.71)^a^
Insulin Lispro (Humalog)	17	Medications for diabetes	1.04 (0.65–1.68)	1.04 (0.03)	1.04 (0.7)	0.06 (−0.62)
Clopidogrel	25	Platelet coagulation inhibitor	1.94 (1.31–2.88)^a^	1.94 (11.38)	1.94 (1.4)	0.95 (0.39)^a^
Interferon beta-1a (Avonex)	453	β-Interferon	9.43 (8.57–10.38)^a^	9.42 (3151.99)^a^	8.78 (8.11)^a^	3.13 (2.99)^a^
Botulinum toxin type A (Botox)	22	Other medications	1.3 (0.85–1.97)	1.3 (1.49)	1.3 (0.91)	0.37 (−0.23)
Pamidronate Disodium (Aredia)	14	Other medications	2.42 (1.43–4.08)^a^	2.42 (11.59)^a^	2.41 (1.56)	1.27 (0.52)^a^

^a^ Indicates that the algorithm is meaningful.

### 3.3 Classification of drugs based on risk level

**FIGURE 4:**
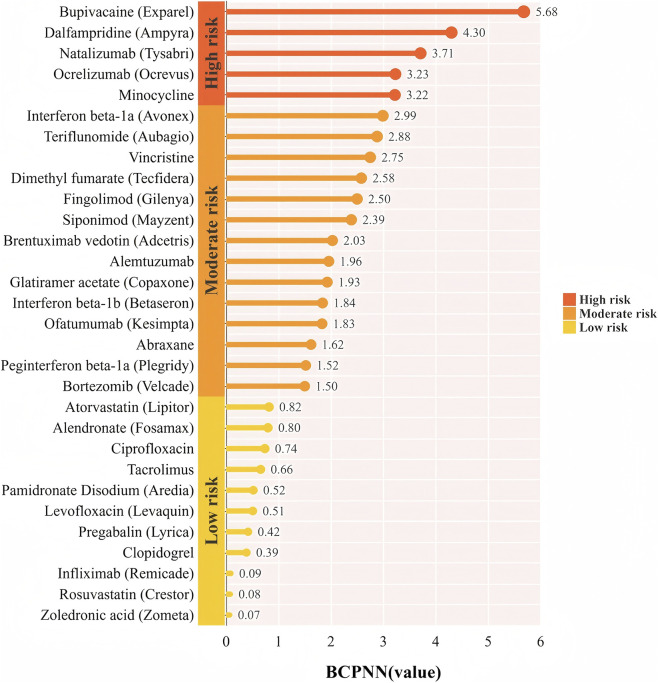
Ranking of drug risk levels using the BCPNN algorithm.

**FIGURE 5:**
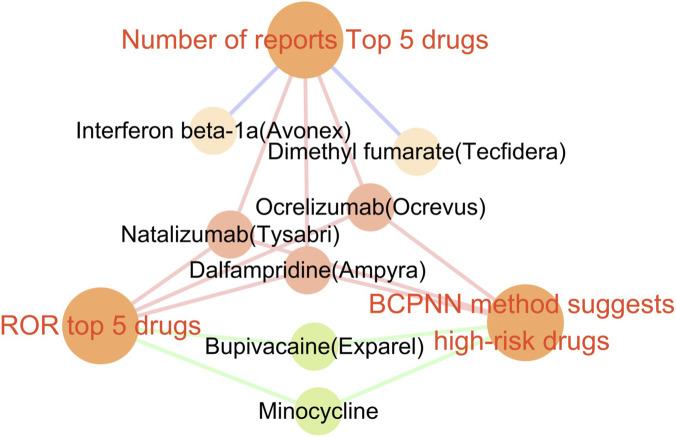
The relationship among the number of reports for the Top 5 drugs, the ROR for the Top 5 drugs, and the BCPNN method indicates the identification of high-risk drugs.

To further evaluate drug-related risks, the BCPNN algorithm was employed. This method classifies risk levels based on predefined thresholds: BCPNN values ranging from 0 to 1.5 indicate a low risk of drug-related adverse events, values between 1.5 and 3 suggest a moderate risk, and values exceeding 3 signify a high risk. Utilizing this approach, we identified the high-risk drugs as follows: bupivacaine (5.68), dalfampridine (4.3), natalizumab (3.71), ocrelizumab (3.23) and Minocycline (3.22) (Figure 4 provides a detailed ranking of these drugs), indicating a significant association with an elevated risk of peripheral nerve palsy. Furthermore, the ROR analysis method also reached a similar results. These findings indicate the necessity for more rigorous monitoring and comprehensive assessment of these high-risk medications within a clinical environment. Figure 5 illustrates the association among the top five drugs based on the number of adverse event reports, the top five drugs identified by the ROR, and the high-risk drugs highlighted by the BCPNN method (Figure 5).

## 4 Discussion

Peripheral nerve palsy significantly impairs patients’ quality of life and may lead to disability in severe cases. Given the irreversible nature of nerve damage, the therapeutic outcomes for most types of nerve palsy remain suboptimal. Therefore, promptly identifying the underlying cause of nerve palsy is crucial for accurate diagnosis and effective treatment. Research indicates that certain medications can lead to peripheral nerve palsy. In this study, we conducted a comprehensive analysis of drug-associated adverse events related to peripheral nerve palsy, utilizing data reported in the FAERS database from January 2004 through the third quarter of 2024.

### 4.1 Analysis of research data

Our findings indicate a noticeable upward trend in the number of reports over time, which then stabilizes, suggesting an increasing awareness of peripheral nerve palsy and enhancements in reporting systems in recent years. Case reports of peripheral nerve palsy are predominantly documented in developed countries, potentially reflecting disparities in healthcare infrastructure and development between developed and developing regions. The study also found that among patients experiencing drug-induced peripheral nerve palsy, females were more numerous than males across all age groups, with a female-to-male ratio approaching 2:1. The age groups most commonly affected ranged from 41 to 70 years. Notably, the majority of these cases were associated with medications primarily used to treat multiple sclerosis (MS), which may be linked to the well-established gender disparity in MS itself—where the female-to-male ratio is also approximately 2:1. Additionally, the cumulative incidence of MS was observed to be higher within the 41–70-year-old population. Regarding data sources, 42.54% of the case information was submitted by healthcare professionals, while fewer reports included patient weight data. Of particular concern is the notably high proportion of cases involving hospitalisation-initial or prolonged and other serious conditions, which underscores the considerable impact that peripheral nerve palsy has on affected patients.

### 4.2 Drugs associated with peripheral nerve palsy

Using four methods of disproportionality analysis, we identified 30 drug-associated peripheral nerve palsies with positive signals, which included a wide range of drug classes that showed significant differences in the risk associated with inducing peripheral nerve palsy, and of particular note, drug classifications that included three or more classes that can cause peripheral nerve palsy included Antineoplastic drugs and immunosuppressants, immunosuppressant, Immunomodulatory drugs, antitumor drugs, Systemic anti-infective drugs. Among the drugs reported in high numbers were natalizumab, interferon beta-1a, dalfampridine, dimethyl fumarate, ocrelizumab, which, interestingly, are all drugs for the treatment of MS, mostly immunosuppressants, biological preparation. Drugs closely associated with an increased risk of peripheral nerve palsy were bupivacaine, dalfampridine, natalizumab, ocrelizumab, and minocycline. These findings provide valuable real-world data and theoretical support for clinical decision-making, guide the rational use of medications to reduce the risk of peripheral nerve palsy, and provide important insights for future research.

#### 4.2.1 Drugs for the treatment of multiple sclerosis (MS) and peripheral nerve palsy

The drugs most commonly linked to the development of peripheral nerve palsy are those used in the management of MS. Notably, natalizumab has been frequently reported in association with this adverse effect. Additionally, other MS therapies such as interferon beta-1a, dalfampridine, and ocrelizumab are also closely associated with an increased risk of peripheral nerve palsy. MS, an immune-mediated neurodegenerative disorder affecting the central nervous system (CNS), is characterized by the formation of inflammatory lesions and demyelinating plaques. As the disease advances, it leads to irreversible axonal injury, contributing to progressive neurological decline (Dobson and Giovannoni, 2019). The first drug, natalizumab, a monoclonal antibody directed against the alpha chain of the VLA-4 integrin, is a potent inhibitor of cell migration toward the tissues including CNS. It potently reduces relapses and active brain lesions in the relapsing remitting form of the disease (Khoy et al., 2020). Natalizumab is currently used to treat relapsing-remitting MS (Outteryck, 2016). Its primary objectives are to decrease the frequency of clinical exacerbations, reduce the number and volume of active lesions identified on magnetic resonance imaging (MRI) scans, and slow the progression of physical disability associated with the disease (Morrow et al., 2022). The most frequently observed adverse reactions to date include headache, infections, and joint pain (Rodríguez de Castro et al., 2021). In the nervous system, the main adverse reactions mentioned were vertigo and narcolepsy. The drug is also used to treat Crohn’s disease (Nelson et al., 2018). The second drug, interferon beta-1a, is among the primary therapies for patients with MS. In addition to its efficacy in decreasing the frequency of relapses, it also impacts the disease’s progression by mitigating the accumulation of lasting disability (Cohan et al., 2021). In the MS Collaborative Research Group, IM IFN β-1a reported a significantly higher frequency of AEs than placebo patients and was limited to flu-like symptoms, muscle aches, chills, fever and weakness (Jacobs et al., 1996). The most frequently reported AEs associated with interferon beta-1a in a 6-year study included flu-like symptoms, headache, myalgia, cold-like symptoms, and accidental injuries (Herndon et al., 2005). The third drug is dalfampridine, a potassium channel blocker approved to improve walking in people with MS (Hupperts et al., 2016; Pikoulas and Fuller, 2012). Dalfampridine, in the specification, has identified adverse reactions mainly neurological reactions, including convulsive attacks, insomnia, balance disorders, dizziness, headache and malaise, and one of the most frequent adverse reactions is urinary tract infections. Additionally, numerous studies have further corroborated the existence of these adverse reactions (Hupperts et al., 2016; Hobart et al., 2019; Yapundich et al., 2015; Goodman et al., 2010; De Giglio et al., 2019; Arreola-Mora et al., 2019; Zhang et al., 2021). The fourth drug, ocrelizumab, is a humanized anti-CD20 monoclonal antibody administered via intravenous infusion. It has received approval for the treatment of adult patients with relapsing forms of MS as well as primary progressive multiple sclerosis (Lamb, 2022). The adverse reactions detailed in the ocrelizumab insert primarily encompass infusion-related reactions such as pruritus, rash, urticaria, erythema, bronchospasm, and vertigo. Additionally, the profile includes an increased risk of infections, including upper and lower respiratory tract infections, as well as herpes-related infections. Malignant diseases, notably breast cancer, are also listed among potential adverse events (Gelfand et al., 2017).

Interestingly, the above drug instructions do not mention nerve palsy-related adverse reactions, which may be attributable to the fact that MS itself can cause nerve palsy symptoms that resemble adverse drug effects. Non-medical professionals might inadvertently classify these MS symptoms as adverse reactions, leading to a higher reported incidence. To clarify whether these drugs truly cause nerve palsy, further investigation through rigorous professional studies is required to provide definitive evidence.

#### 4.2.2 Anaesthetics and peripheral nerve palsy

BCPNN suggests that bupivacaine (Exparel) is strongly associated with an increased risk of peripheral nerve palsy. Bupivacaine is a amide local anesthetic that exerts local anesthetic and analgesic effects by blocking voltage-gated sodium channels and inhibiting the generation and conduction of action potentials. Exparel is a multivesicular liposomal formulation of bupivacaine (Ilfeld et al., 2015). It is primarily intended for single-dose administration directly into the surgical site to deliver effective postoperative pain relief. Additionally, it can be utilized in adult patients for interosseous groove brachial plexus nerve blocks, thereby providing targeted regional analgesia following surgery. The most common adverse reactions (incidence >10%) following bupivacaine administration were found to be nausea, constipation and vomiting (Ilfeld et al., 2015). In studies involving the surgical site infiltration administration of bupivacaine, the neurological adverse reactions categorized by system organ class included dizziness, headache, somnolence, hyperalgesia, and drowsiness. Signs and symptoms of bupivacaine overdose encompass both CNS effects—such as perioral sensory abnormalities, dizziness, dysarthria, and sensory and visual disturbances—and cardiovascular system adverse effects, including myocardial depression, hypotension, bradycardia, and cardiac arrest (Aggarwal, 2018; Tong et al., 2014). We speculate that susceptibility factors for bupivacaine-induced neurotoxicity may be associated with variables such as the dosage administered, the specific site of injection, and the duration of exposure. Additionally, intraoperative factors like positional traction or compression, local ischemia, and hypoxia, as well as an acidic microenvironment, can potentially diminish the nerve’s capacity to withstand the effects of local anesthetics and exacerbate their neurotoxic outcomes. Underlying metabolic or microenvironmental disturbances, such as diabetes mellitus, might further worsen local anesthetic-related mitochondrial oxidative stress and neuroinflammatory responses, thereby increasing the vulnerability of peripheral nerves to injury (Ji et al., 2017). Clinical observations have noted that some patients may develop nerve palsy following nerve block anesthesia (Shlizerman and Ashkenazi, 2005); however, there is currently no detailed research elucidating the underlying mechanisms. This complication may be attributed to neurotoxicity induced by the anesthetic agents, but definitive evidence and a comprehensive understanding of the specific pathophysiological processes remain to be established through further investigation in future studies.

Studies have shown that the pathophysiological mechanism by which bupivacaine causes neurotoxicity may include: endoplasmic reticulum stress response (Liu et al., 2019), activation of the PI3K and MAPK signaling pathways (Lirk et al., 2008; Zhao et al., 2016), abnormal autophagy (Wang et al., 2019), increased translocation of the p47phox membrane, leading to excessive production of reactive oxygen species and neuronal apoptosis (Li et al., 2017).

Since bupivacaine has the potential to cause severe adverse effects, it is essential to exercise caution regarding its dosage and the selection of the appropriate injection site. Following the administration of bupivacaine, continuous and close monitoring of the patient’s vital signs and level of consciousness is imperative. Additionally, the patient should be carefully observed for signs of nerve palsy post-injection to promptly identify and manage any adverse reactions and minimize associated risks.

#### 4.2.3 Antibiotics and peripheral nerve palsy

Peripheral nerve damage attributed to antibiotic use is relatively common in clinical practice, particularly with anti-tuberculosis medications (Arsalan and Sabzwari, 2015; Shetty and Shah, 2019) and fluoroquinolones (Refaeian et al., 2023; Popescu, 2018; Cohen, 2001). The BCPNN method indicates a strong association between minocycline and an increased risk of peripheral nerve palsy. Minocycline, a second-generation tetracycline derivative (Singh et al., 2021), is widely used in the treatment of acne vulgaris (Onge and Mobley, 2021; Garner et al., 2012). Reports have documented cases where minocycline induces multiplex mononeuritis, with discontinuation leading to clinical improvement in affected patients (Kang et al., 2018). Other studies have found that typical neuropathy associated with minocycline use is painful single or multiple mononeuropathy due to peripheral nerve vasculitis. These studies concluded that minocycline has a promoting effect on peripheral nerve palsy (Baratta et al., 2015; Ogawa et al., 2010). However, contrasting evidence exists; some studies demonstrate that minocycline can prevent the development of mechanical allodynia in mouse models of vincristine-induced peripheral neuropathy (Starobova et al., 2019), and systemic administration of minocycline has been shown to mitigate intrafascicular lidocaine-induced peripheral nerve damage (Cheng et al., 2016). Furthermore, minocycline appears to attenuate diabetic neuropathy by inhibiting spinal cord Notch signaling in rat models (Yang et al., 2017). These studies have indicated that minocycline may exert a beneficial effect in the management of nerve injury. However, given the conflicting results reported in previous research, further investigations are essential to definitively establish whether minocycline can induce nerve palsy and to elucidate the underlying mechanisms involved.

#### 4.2.4 Antineoplastic drugs and peripheral nerve palsy

This study found that antineoplastic drugs can cause nerve palsy, the main drugs found include vincristine, bortezomib, brentuximab vedotin, and abraxane, which are suggested to be associated with a moderate risk of peripheral nerve palsy by the BCPNN method. Chemotherapy-induced peripheral neuropathy is a common and relevant side effect of antineoplastic agents such as abraxane, vincristine and bortezomib (Bobylev et al., 2015; Stage et al., 2021; Chen X. et al., 2024).

Numerous reports have documented neurological damage induced by antineoplastic drugs. Neuropathy may manifest as sensory, motor, and/or autonomic dysfunction, leading to interruptions, delays, or discontinuation of chemotherapy. These neurological impairments can cause significant pain, disability, and a decline in the patient’s quality of life, in addition to increased healthcare costs associated with managing these complications (Triarico et al., 2021). The relevant mechanism of action may be described as follows: Vincristine primarily exerts its effects by disrupting the normal processes of microtubule assembly and disassembly. This interference leads to a blockade in mitosis, ultimately resulting in cell death. Additionally, vincristine induces alterations in the morphology and structural integrity of neurons, impairing both retrograde and anterograde axonal transport. These disruptions contribute to Wallerian degeneration. Furthermore, vincristine affects the transmission of nerve impulses and leads to neuronal death, thereby resulting in peripheral nerve damage (Triarico et al., 2021; Starobova and Vetter, 2017). Although bortezomib is unable to penetrate the CNS due to the blood-brain barrier, it accumulates in the dorsal root ganglion (DRG) and causes neurotoxicity. Upon accumulation in the DRG, bortezomib indirectly contributes to CNS dysfunction, including neuroglial activation, disruption of glutamatergic homeostasis, and inflammation (Meregalli et al., 2014; Yamamoto and Egashira, 2021).

Brentuximab vedotin is an anti-CD30 monoclonal antibody-drug coupling approved for the treatment of relapsed or refractory Hodgkin’s lymphoma (Matthys et al., 2024; Oak and Bartlett, 2016). Peripheral neurotoxicity is a type of off-target toxicity of brentuximab vedotin and represents the most common extra-haematological and the main clinically significant brentuximab vedotin-related toxicity (Velasco et al., 2021). Studies have shown that brentuximab vedotin-induced inflammatory neuropathies can appear in patients treated with brentuximab vedotin, often presenting as sensory abnormalities, muscle weakness and gait disorders (Matthys et al., 2024). Severe peripheral neuropathy with significant motor deficits was reported in approximately 10% of patients in the brentuximab vedotin pivotal clinical trial (Farge et al., 2020). The mechanism by which brentuximab vedotin causes nerve palsy may be: microtubules are important components of nerve axons and play a key role in maintaining axonal transport between neuronal cell bodies and distal nerve endings. The component of brentuximab vedotin contains the antitubulin agent monomethyl auristatin E (MMAE). MMAE binds extensively to tubulin and microtubule and causes severe microtubule dysregulation via blockade of tubulin polymerisation damages the axon structure and inhibits axon transport, thereby damaging peripheral nerves and leading to peripheral nerve palsy (Velasco et al., 2021).

Abraxane is widely used to treat common cancers such as breast, ovarian and lung cancer. Although abraxane is very effective in blocking tumor progression, it also causes peripheral neuropathy in 60%–70% of chemotherapy patients. The mechanism by which abraxane causes peripheral nerve palsy may be related to its ability to induce altered calcium signaling, neuropeptide and growth factor release, mitochondrial damage and reactive oxygen species formation, and can activate ion channels that mediate responses to extracellular cues (Staff et al., 2020; Vermeer et al., 2021).

In conclusion, our study is similar to previous studies in that antineoplastic drugs are correlated with peripheral nerve palsy.

#### 4.2.5 Other drugs and peripheral nerve palsy

Our study further identified that medications associated with peripheral nerve palsy encompass drugs acting on the musculoskeletal system, analgesics, lipid-modulating agents, platelet aggregation inhibitors, and other drug categories. These medications necessitate careful monitoring during clinical application. The appearance of adverse events suggestive of peripheral nerve palsy warrants immediate evaluation. If such a diagnosis is confirmed, the discontinuation or reduction of the suspected medication should be carefully considered, taking into account the patient’s overall clinical status and guided by sound clinical judgment.

## 5 Limitations

From a pharmacovigilance perspective, although our study analysed drugs with a potential risk of inducing peripheral nerve palsy events, there are inherent limitations to the study. First, FAERS is based on a self-reporting system that carries the risk of underreporting, duplicate reporting, and inaccurate reporting. Despite our deduplication, the study results may still be biased. Second, various factors such as patient age, drug interactions, dosage and duration of drug use, and comorbidities can affect the occurrence of peripheral nerve palsy and the results of the analyses. Finally, the results of the signalling assay for peripheral nerve palsy only suggest a statistical correlation between the drug of interest and the target AE report, and further in-depth studies are needed to identify specific mechanisms and establish a clear causal relationship.

## 6 Conclusion

The data collected and analyzed in our study indicate that a wide range of medications—including immunosuppressants, biological preparation, anesthetics, antibiotics, and antineoplastic drugs—are linked to the incidence of peripheral nerve palsy. Notably, specific drugs such as natalizumab, dalfampridine, ocrelizumab, bupivacaine, and minocycline have been particularly associated with this condition. Clinicians should carefully weigh not only the therapeutic benefits of these medications but also their potential AEs to reduce both the occurrence and severity of peripheral nerve palsy. Furthermore, large-scale clinical trials are crucial to confirm these findings, uncover the underlying mechanisms of drug-induced peripheral nerve palsy, and refine clinical guidelines accordingly.

## Data Availability

Publicly available datasets were analyzed in this study. This data can be found here: FDA Adverse Event Reporting System, https://www.fda.gov/drugs/surveillance/fdas-adverse-event-reporting-system-faers.
